# Association of Sperm Preparation and Fertilization Media Combinations With Fertilization Outcomes in Conventional In Vitro Fertilization: A Single-Center Retrospective Study

**DOI:** 10.7759/cureus.110549

**Published:** 2026-06-09

**Authors:** Mitsuru Nago, Manon Koyata, Yuria Takahashi, Akari Saito, Eri Kamioka, Bunpei Ishizuka

**Affiliations:** 1 Embryology Laboratory, Assisted Reproductive Technology, Rose Ladies Clinic, Tokyo, JPN; 2 Department of Obstetrics and Gynecology, Rose Ladies Clinic, Tokyo, JPN

**Keywords:** conventional ivf, fertilization medium, glucose, medium ph, normal fertilization, short gamete co-incubation, sperm preparation medium

## Abstract

Objective

Short gamete co-incubation is used in conventional in vitro fertilization (IVF) protocols to reduce the duration of oocyte-sperm exposure and enable earlier identification of fertilization failure. However, laboratory conditions associated with fertilization outcomes under short co-incubation remain insufficiently characterized. This retrospective study evaluated whether different combinations of sperm preparation and fertilization media were associated with fertilization outcomes during short gamete co-incubation in conventional IVF.

Methods

This single-center retrospective study screened 216 insurance-covered conventional IVF cycles comprising 1,591 oocytes performed between August 2023 and July 2025. After application of exclusion criteria, 738 mature oocytes from 108 cycles were included in the analysis. Mature oocytes were defined as those exhibiting at least one polar body after IVF. Cycles were categorized into three medium-combination groups according to the laboratory protocol period in which each combination was used: Group A (Iso × Gx-IVF; January 2024 to March 2025), Group B (Gra × Gx-IVF; August to November 2023), and Group C (Gra × SF; April to July 2025), with December 2023 treated as an excluded mixed-medium transition period. Outcomes were analyzed per mature oocyte. The primary outcome was two-pronuclear (2PN) fertilization. Secondary outcomes included 0PN, 1PN, ≥3PN, and the proportion of good-quality embryos on Day 2. Multivariable logistic regression models with cycle-clustered robust standard errors were used to account for intra-cycle correlation among oocytes.

Results

The 2PN fertilization rate differed significantly among the three groups: 43.0% in Group A, 59.8% in Group B, and 66.9% in Group C (p < 0.001). The 0PN rate was highest in Group A and differed significantly among groups: 49.8% in Group A, 30.8% in Group B, and 21.5% in Group C (p < 0.001). The proportion of good-quality embryos on Day 2 was 33.2%, 41.9%, and 42.1% in Groups A, B, and C, respectively; however, this difference did not reach statistical significance (p = 0.067). In cycle-clustered multivariable logistic regression models, Group B was associated with significantly higher odds of 2PN fertilization than Group A after adjustment for patient characteristics, semen parameters, and time-related laboratory factors (adjusted odds ratio (aOR), 2.307; 95% confidence interval (CI), 1.165-4.572; p = 0.017). Group C showed a similar trend toward higher odds of 2PN fertilization, although the association was borderline after full adjustment (aOR, 2.738; 95% CI, 1.001-7.484; p = 0.050).

Conclusion

In this single-center retrospective study, sperm preparation and fertilization medium-combination protocols were associated with 2PN and 0PN fertilization outcomes during short gamete co-incubation in conventional IVF. Compared with Group A, Group B demonstrated higher adjusted odds of 2PN fertilization after controlling for patient characteristics, semen parameters, and time-related laboratory factors, whereas Group C showed a similar trend with borderline statistical significance after full adjustment. These findings are limited to fertilization outcomes and should be interpreted as hypothesis-generating because of the retrospective design, non-randomized implementation across different calendar periods, and lack of downstream reproductive outcomes. Prospective studies are warranted to confirm these observations.

## Introduction

Conventional in vitro fertilization (IVF) remains an important insemination method because it allows oocytes and spermatozoa to interact without micromanipulation. However, prolonged gamete co-incubation has been suggested to increase oxidative stress within the culture environment. In a mouse model, prolonged exposure of embryos to spermatozoa resulted in elevated levels of reactive oxygen species in the fertilization medium and was associated with impaired embryo development [1]. On this basis, short gamete co-incubation has been adopted in selected IVF laboratories to limit the duration of oocyte-sperm exposure. It may also facilitate earlier identification of fertilization failure, including consideration of early rescue intracytoplasmic sperm injection (ICSI) in selected cases [2]. However, the reported effects of short co-incubation on fertilization and embryo development remain inconsistent [3,4].

Under short co-incubation conditions, the laboratory environment during sperm preparation and fertilization may be particularly relevant, as spermatozoa must acquire and maintain fertilizing capacity within a limited exposure period. In this context, pH and energy substrates are of particular interest. Sperm motility and capacitation are closely linked to pH regulation. Experimental studies have shown that acidic pH impairs human sperm motility and capacitation and that capacitation is accompanied by intracellular alkalinization in human spermatozoa [5,6]. The pH of the human female reproductive tract varies according to anatomical site, menstrual phase, and measurement conditions. Previous studies have described site-specific and cycle-related variation in reproductive tract pH and emphasized the relevance of pH regulation to sperm function [7,8]. These observations suggest that mildly alkaline environments may be physiologically relevant to sperm function; however, the direct clinical implications of using a sperm preparation medium with a higher pH than standard media in IVF remain unclear. In addition, glucose is a key metabolic substrate for sperm function. Previous studies have reported that glucose supports human sperm motility and capacitation and that its presence in the medium is required for sperm penetration of the human zona pellucida in experimental models [9,10]. Collectively, these findings suggest that the combination of sperm preparation and fertilization media may be associated with fertilization outcomes in conventional IVF; however, clinical evidence evaluating such combinations remains limited.

In our laboratory, three different combinations of sperm preparation and fertilization media were used during distinct laboratory protocol periods while short gamete co-incubation was routinely performed. Among analyzable cycles, the implementation sequence was Group B, followed by Group A and then Group C, with December 2023 treated as an excluded mixed-medium transition period. Group A was used as the analytical reference category because it represented the largest and most stable routine protocol period in the present dataset. The details of each medium-combination group are provided in the Materials and Methods section. Because these combinations were introduced as part of routine laboratory practice rather than as a randomized intervention, their associations with fertilization outcomes warrant careful retrospective evaluation with adjustment for patient characteristics, semen parameters, and time-related laboratory factors. Therefore, this single-center retrospective study aimed to investigate whether different combinations of sperm preparation and fertilization media were associated with fertilization outcomes per mature oocyte in conventional IVF under short gamete co-incubation conditions.

## Materials and methods

Study design and ethical approval

This single-center retrospective cohort study included conventional IVF cycles performed at our clinic. The study period extended from August 2023 to July 2025, during which insurance-covered conventional IVF cycles using freshly ejaculated sperm were screened for eligibility.

Eligible cycles were categorized into three groups according to the laboratory protocol period during which each combination of sperm preparation and fertilization media was used. Group B (Gra × Gx-IVF) was used from August to November 2023, Group A (Iso × Gx-IVF) was used from January 2024 to March 2025, and Group C (Gra × SF) was used from April to July 2025. Cycles performed in December 2023 were treated as a mixed-medium transition period and were excluded from group classification when the applied medium-combination information was not recorded in an analyzable format. These medium combinations were not introduced as a randomized intervention but were implemented as part of routine laboratory practice changes.

This study was conducted in accordance with the Declaration of Helsinki and was approved by the Rose Ladies Clinic Ethics Committee (approval number: RLC-050). The requirement for written informed consent was waived by the Ethics Committee because of the retrospective nature of the study and the use of anonymized clinical data. The study protocol was published on the clinic website, and an opt-out approach was adopted to allow patients to decline participation. The data used for analysis were anonymized and handled in a manner that prevented personal identification.

Cycle and oocyte selection

**Figure 1:**
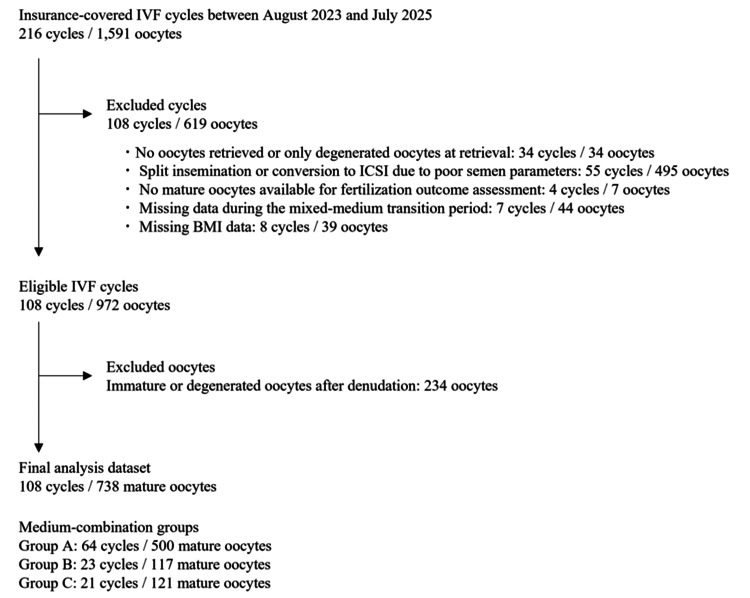
Flow diagram of cycle and oocyte selection A total of 216 insurance-covered conventional in vitro fertilization (IVF) cycles comprising 1,591 oocytes, performed between August 2023 and July 2025, were screened for eligibility. After cycle-level and oocyte-level exclusions, 738 mature oocytes from 108 cycles were included in the final analysis. The final analysis dataset was categorized into three medium-combination groups: Group A (Iso × Gx-IVF; January 2024 to March 2025), Group B (Gra × Gx-IVF; August to November 2023), and Group C (Gra × SF; April to July 2025). December 2023 was treated as a mixed-medium transition period; cycles during this period were excluded from group classification when the applied sperm preparation or fertilization medium could not be classified. ICSI: intracytoplasmic sperm injection; BMI: body mass index; Iso: ISolate Stock Solution; Gra: ORIGIO Gradient 90; Gx-IVF: Gx-IVF fertilization medium; SF: Sequential Fert The symbol × denotes the combination of sperm preparation medium and fertilization medium.

The flow of cycle and oocyte selection is shown in Figure 1. A total of 216 insurance-covered conventional IVF cycles comprising 1,591 oocytes, performed between August 2023 and July 2025, were screened for eligibility.

At the cycle level, 108 cycles comprising 619 oocytes were excluded. The reasons for exclusion were as follows: no oocytes retrieved or only degenerated oocytes at retrieval (34 cycles/34 oocytes), split insemination or conversion to ICSI due to poor semen parameters (55 cycles/495 oocytes), no mature oocytes available for fertilization outcome assessment (4 cycles/7 oocytes), unclassifiable medium-combination exposure during the December 2023 mixed-medium transition period because the applied medium-combination information was not recorded in an analyzable format (7 cycles/44 oocytes), and missing body mass index (BMI) data (8 cycles/39 oocytes). During the December 2023 mixed-medium transition period, the applied sperm preparation or fertilization medium was not consistently recorded in an analyzable format for some cycles. These cycles were excluded to avoid misclassification of medium-combination exposure.

After cycle-level exclusions, 108 cycles comprising 972 oocytes remained eligible. Among these, 234 oocytes assessed as immature or degenerated at denudation after IVF were excluded at the oocyte level. Finally, 738 mature oocytes from 108 cycles were included in the analysis. Mature oocytes were defined as those with at least one polar body observed at denudation after IVF.

Medium-combination groups

The included cycles were categorized into three groups according to the combination of sperm preparation and fertilization media used during each laboratory protocol period. Among analyzable cycles, the implementation sequence was Group B, followed by Group A and then Group C, with December 2023 treated as an excluded mixed-medium transition period. For clarity of statistical presentation, the groups are presented as Group A, Group B, and Group C according to the analytical reference structure rather than chronological order. Group A was selected as the reference category because it represented the largest and most stable routine protocol period in the present analysis dataset; this choice does not imply chronological priority, random allocation, or causal superiority.

In Group A (Iso × Gx-IVF), used from January 2024 to March 2025, a 90% density gradient prepared from ISolate Stock Solution (FUJIFILM Wako Pure Chemical Corporation, Osaka, Japan) was used as the sperm preparation medium, and Gx-IVF (Vitrolife, Gothenburg, Sweden) was used as the fertilization medium.

In Group B (Gra × Gx-IVF), used from August to November 2023, ORIGIO Gradient 90 (CooperSurgical, Inc., Trumbull, CT, USA) was used as the sperm preparation medium, and Gx-IVF was used as the fertilization medium.

In Group C (Gra × SF), used from April to July 2025, ORIGIO Gradient 90 was used as the sperm preparation medium, and Sequential Fert medium (CooperSurgical, Inc.) was used as the fertilization medium.

The pH of the sperm preparation media was measured at 24°C under air-equilibrated conditions. The pH values of the 90% density gradient prepared from ISolate Stock Solution and ORIGIO Gradient 90 were 7.395 ± 0.001 and 8.061 ± 0.003, respectively. Values are presented as mean ± standard deviation based on single measurements obtained from three different lots.

Because glucose concentrations in the fertilization media were not directly measured in the present study, they were treated as reported medium characteristics derived from previous literature. Zagers et al. reported that G-IVF and G-IVF PLUS contained approximately 2.51-2.56 mM glucose, whereas ORIGIO Sequential Fert contained 5.01 mM glucose [11]. Although Gx-IVF was not directly listed in the supplementary table reported by Zagers et al., Mizumoto et al. stated that Gx-series media and G-series media have identical compositions except for antioxidant supplementation [12]. Therefore, in the present study, the glucose concentration of Gx-IVF was interpreted with reference to the reported values for G-series fertilization media. This study was designed as a retrospective clinical analysis of the association between medium combinations and fertilization outcomes in routine practice rather than as an experimental comparison of individual medium components.

Ovarian stimulation and oocyte retrieval

Ovarian stimulation was performed according to ovarian reserve, patient characteristics, and physician judgment. The stimulation protocols included gonadotropin-releasing hormone (GnRH) agonist protocols, GnRH antagonist protocols, GnRH antagonist protocols combined with letrozole, clomiphene citrate plus human menopausal gonadotropin (hMG), follicle-stimulating hormone (FSH)-based stimulation with or without hMG, progestin-primed ovarian stimulation (PPOS), and natural-cycle IVF.

The timing of oocyte retrieval was determined based on the diameter of the leading follicle, serum hormone levels, and clinical judgment. Final oocyte maturation was triggered using human chorionic gonadotropin (hCG) or a GnRH agonist. Transvaginal ultrasound-guided oocyte retrieval was performed approximately 36-37 hours after triggering.

Sperm examination and sperm preparation

Semen samples were collected by masturbation after patients were instructed to maintain an abstinence period of 2-7 days. However, the actual duration of abstinence was not recorded in an analyzable format in the medical records and, therefore, was not included in the present analysis. Semen samples were allowed to liquefy at 24°C for at least 15 minutes. After liquefaction, semen volume was measured using a graduated syringe. Sperm concentration and motility were assessed by experienced embryologists using microscopy with a Makler sperm counting chamber (Sefi Medical Instruments, Haifa, Israel).

The total motile sperm count per ejaculate was calculated using the following formula:

\(\text{Total motile sperm count per ejaculate} = \frac{\text{semen volume (mL)} \times \text{sperm concentration }(\times 10^{6}/\text{mL}) \times \text{progressive motility }(\%)}{100}\)

Sperm preparation for IVF was performed using density gradient centrifugation combined with a swim-up procedure. Liquefied semen was layered onto 3.0 mL of density gradient medium. A 90% density gradient prepared from ISolate Stock Solution was used in Group A, whereas ORIGIO Gradient 90 was used in Groups B and C. The sample was centrifuged at 400 × g for 15 minutes. After centrifugation, the sperm pellet was resuspended in a group-specific sperm wash medium: MHM handling medium (FUJIFILM Wako Pure Chemical Corporation) for Group A and ORIGIO Sperm Wash (CooperSurgical, Inc.) for Groups B and C.

A volume of 3.0 mL of the corresponding wash medium was added, and the sample was centrifuged at 200 × g for five minutes. After removal of the supernatant, 0.2 mL of the same wash medium was gently layered over the sperm pellet. The tube was tilted to approximately 45° and left undisturbed at 24°C for 15 minutes to allow motile spermatozoa to swim up. The upper 0.1 mL of the medium was carefully aspirated and transferred to a sterile tube. Sperm concentration and motility were then reassessed, and the prepared motile spermatozoa were used for insemination.

IVF procedure and embryo culture

Cumulus-oocyte complexes recovered after oocyte retrieval were placed in the fertilization medium assigned to each group: Gx-IVF for Groups A and B and ORIGIO Sequential Fert for Group C. The volume of fertilization medium was 1 mL, and one to five cumulus-oocyte complexes were placed in each 1 mL drop. The fertilization medium was overlaid with HiGROW OIL Heavy (Fuso Pharmaceutical Industries, Ltd., Osaka, Japan) and pre-equilibrated at 37°C under humidified conditions of 6% CO_2_, 5% O_2_, and 89% N_2_ using an APM-30D humidified incubator (ASTEC Co., Ltd., Fukuoka, Japan).

Prepared motile spermatozoa were added to the fertilization medium to achieve a final concentration of 1 × 10^5^ motile spermatozoa/mL. The laboratory protocol was designed for short gamete co-incubation with an intended duration of approximately 240 minutes. However, the actual gamete co-incubation time ranged from 233 to 302 minutes due to the routine laboratory workflow. Therefore, the actual co-incubation time was recorded for each oocyte and included as a time-related covariate in the multivariable analysis.

After gamete co-incubation, cumulus cells were removed by mechanical pipetting. Oocytes with at least one polar body observed at denudation after IVF were defined as mature oocytes. Fertilization and subsequent embryo development outcomes were calculated using these mature oocytes as the denominator. Mature oocytes were transferred to Gx-TL (Vitrolife), and embryo culture was continued using a CCM-iBIS-L W10 time-lapse incubator (ASTEC Co., Ltd.) under the same gas conditions (37°C, 6% CO_2_, and 5% O_2_). General culture conditions and handling procedures were standardized across groups, except for the sperm preparation and fertilization media used during each laboratory protocol period.

Fertilization and Day 2 embryo assessment

Fertilization status was assessed on the day after insemination (Day 1). Oocytes with two pronuclei were classified as normally fertilized (two-pronuclear (2PN)). Oocytes without pronuclei were classified as 0PN. Abnormal fertilization was defined as the presence of 1PN or ≥3PN.

Cleavage-stage embryos were evaluated morphologically on Day 2. Embryo morphology was assessed according to the Veeck classification [13]. In the present study, based on institutional criteria, good-quality embryos on Day 2 were defined as embryos with Veeck grades 1-3 and 3-5 blastomeres. The Day 2 good-quality embryo rate was calculated per mature oocyte.

Outcomes

The primary outcome was the 2PN fertilization rate per mature oocyte. Secondary outcomes included the rates of 0PN, 1PN, ≥3PN, and good-quality embryos on Day 2 per mature oocyte.

Statistical analysis

Continuous variables are presented as median (interquartile range) and were compared among the three groups using the Kruskal-Wallis test. Categorical variables are presented as n/N (%) and were compared using Fisher’s exact test. When appropriate, pairwise comparisons were conducted with Bonferroni correction.

Multivariable logistic regression models were used to evaluate factors associated with 2PN fertilization. The unit of analysis was the mature oocyte. Because multiple mature oocytes could be obtained from the same treatment cycle, cycle-clustered robust standard errors were applied to account for within-cycle correlation among oocytes.

Medium combination was entered as a categorical variable, with Group A used as the analytical reference category because it represented the largest and most stable routine protocol period in the present analysis dataset. This reference category was selected for statistical interpretability and does not imply chronological priority, random allocation, or causal superiority.

Two models were constructed. Model 1 included medium combination, female age at oocyte retrieval, BMI, male age at oocyte retrieval, number of viable oocytes at retrieval, and total motile sperm count per ejaculate. Model 2 included the variables in Model 1 plus time from semen collection to preparation, oocyte pre-incubation time, and gamete co-incubation time. Total motile sperm count per ejaculate was analyzed per 10 × 10^6^ spermatozoa, and time-related variables were analyzed per 10 minutes.

Because the medium-combination groups were implemented during distinct laboratory protocol periods, the calendar period was closely linked to medium combination and was not included simultaneously in the primary multivariable models to avoid collinearity and unstable estimates. Available time-related laboratory variables were included in Model 2, and residual confounding by calendar time and other unmeasured period-related factors was considered in the interpretation of the results.

As a secondary analysis, the same modeling strategy was applied using 0PN fertilization as the dependent variable. All statistical tests were two-sided, and p-values < 0.05 were considered statistically significant. Statistical analyses were conducted using EZR (version 1.61; Saitama Medical Center, Jichi Medical University, Saitama, Japan), a graphical user interface for R [14].

## Results

Cycle and oocyte selection

A total of 216 insurance-covered conventional IVF cycles comprising 1,591 oocytes, performed between August 2023 and July 2025, were screened for eligibility. At the cycle level, 108 cycles comprising 619 oocytes were excluded. After cycle-level exclusions, 108 cycles comprising 972 oocytes remained eligible. Among these, 234 oocytes assessed as immature or degenerated at denudation after IVF were excluded at the oocyte level. Finally, 738 mature oocytes from 108 cycles were included in the analysis.

The final analysis dataset was categorized into three medium-combination groups according to laboratory protocol period: Group A (Iso × Gx-IVF; January 2024 to March 2025), comprising 64 cycles and 500 mature oocytes; Group B (Gra × Gx-IVF; August to November 2023), comprising 23 cycles and 117 mature oocytes; and Group C (Gra × SF; April to July 2025), comprising 21 cycles and 121 mature oocytes (Figure 1). December 2023 was treated as a mixed-medium transition period, and cycles during this period were excluded from group classification when the applied sperm preparation or fertilization medium could not be classified.

Baseline and laboratory characteristics

Baseline and laboratory characteristics are presented in Table 1. No significant differences were observed among the three groups in female age at oocyte retrieval, male age at oocyte retrieval, BMI, total motile sperm count per ejaculate, estradiol (E2) level on the trigger-decision day, number of viable oocytes at retrieval, or time from semen collection to preparation.

**Table 1:** Baseline and laboratory characteristics of treatment cycles according to sperm preparation and fertilization medium combination Values are presented as median (interquartile range). The number of cycles is shown for each group. p-values were calculated using the Kruskal-Wallis test. Different superscript letters indicate significant pairwise differences based on Bonferroni-adjusted Mann-Whitney U tests. Group A: Iso × Gx-IVF; Group B: Gra × Gx-IVF; Group C: Gra × SF; BMI: body mass index; E2: estradiol; IVF: in vitro fertilization; Iso: ISolate Stock Solution; Gra: ORIGIO Gradient 90; Gx-IVF: Gx-IVF fertilization medium; SF: Sequential Fert In group labels, the symbol × denotes the combination of the sperm preparation medium and fertilization medium.

Variable	Group A (64 cycles)	Group B (23 cycles)	Group C (21 cycles)	p-value
Female age at oocyte retrieval (years)	36.5 (34.0-40.0)	39.0 (32.5-41.0)	38.0 (34.0-39.0)	0.585
Male age at oocyte retrieval (years)	38.5 (35.0-43.0)	39.0 (36.0-43.5)	40.0 (37.0-42.0)	0.914
BMI (kg/m^2^)	20.0 (19.0-22.0)	21.0 (19.5-21.5)	19.0 (19.0-22.0)	0.268
Total motile sperm count per ejaculate (×10^6^ spermatozoa)	71.0 (43.9-114.0)	58.8 (33.0-135.6)	69.0 (55.0-142.0)	0.461
E2 level on the trigger-decision day (pg/mL; capped at 2,000 for analysis)	681.9 (358.8-1,596.0)	688.3 (354.4-1,332.4)	717.6 (370.4-1,964.8)	0.894
Number of viable oocytes at retrieval	5.0 (2.0-14.0)	5.0 (1.0-7.5)	2.0 (1.0-8.0)	0.221
Time from semen collection to preparation (min)	60.5 (26.8-119.3)	78.0 (41.0-103.0)	91.0 (28.0-121.0)	0.785
Oocyte pre-incubation time (min)	130.0^a^ (83.0-164.5)	118.0^a^ (101.0-171.5)	52.0^b^ (33.0-77.0)	<0.001
Gamete co-incubation time (min)	243.5^b^ (240.0-250.0)	247.0^ab^ (241.0-256.5)	253.0^a^ (246.0-283.0)	0.006

Oocyte pre-incubation time differed significantly among the groups (p < 0.001). Group C had a shorter oocyte pre-incubation time than Groups A and B. Gamete co-incubation time also differed significantly among the groups (p = 0.006); in Bonferroni-adjusted pairwise comparisons, Group C had a longer co-incubation time than Group A. These time-related laboratory variables were included in the multivariable models.

Fertilization and early embryo development outcomes

Fertilization and early embryo development outcomes are presented in Table 2. The 2PN fertilization rate differed significantly among the groups: 43.0% in Group A, 59.8% in Group B, and 66.9% in Group C (p < 0.001). In Bonferroni-adjusted pairwise comparisons, Groups B and C had significantly higher 2PN fertilization rates than Group A, whereas no significant difference was observed between Groups B and C.

**Table 2:** Fertilization and early embryo development outcomes according to sperm preparation and fertilization medium combination Values are presented as n/N (%). p-values were calculated using Fisher’s exact test. Different superscript letters indicate significant pairwise differences based on Bonferroni-adjusted Fisher’s exact tests. Mature oocytes were defined as those with at least one polar body observed at denudation after IVF. Day 2 good-quality embryos were defined as embryos with Veeck grades 1-3 and 3-5 blastomeres based on institutional criteria. Group A: Iso × Gx-IVF; Group B: Gra × Gx-IVF; Group C: Gra × SF; n/N: number of oocytes with the outcome/total number of mature oocytes; 2PN: two-pronuclear fertilization; 0PN: absence of pronuclei; 1PN: one-pronuclear fertilization; ≥3PN: three or more pronuclei; IVF: in vitro fertilization; Iso: ISolate Stock Solution; Gra: ORIGIO Gradient 90; Gx-IVF: Gx-IVF fertilization medium; SF: Sequential Fert In group labels, the symbol × denotes the combination of the sperm preparation medium and fertilization medium; ≥ denotes greater than or equal to. The dash indicates that a p-value was not applicable.

Outcome	Group A	Group B	Group C	p-value
Number of mature oocytes	500	117	121	-
2PN	215/500 (43.0)^a^	70/117 (59.8)^b^	81/121 (66.9)^b^	<0.001
0PN	249/500 (49.8)^a^	36/117 (30.8)^b^	26/121 (21.5)^b^	<0.001
1PN	17/500 (3.4)	1/117 (0.9)	3/121 (2.5)	0.392
≥3PN	19/500 (3.8)	10/117 (8.5)	11/121 (9.1)	0.015
Day 2 good-quality embryos	166/500 (33.2)	49/117 (41.9)	51/121 (42.1)	0.067

The 0PN rate also differed significantly among the groups: 49.8% in Group A, 30.8% in Group B, and 21.5% in Group C (p < 0.001). In Bonferroni-adjusted pairwise comparisons, Groups B and C had significantly lower 0PN rates than Group A, whereas no significant difference was observed between Groups B and C. The 1PN rate did not differ significantly among the groups. The ≥3PN rate differed significantly in the overall comparison; however, no significant pairwise differences were observed after Bonferroni correction. The Day 2 good-quality embryo rate was 33.2% in Group A, 41.9% in Group B, and 42.1% in Group C; however, this difference did not reach statistical significance (p = 0.067).

Multivariable analysis for 2PN fertilization

Multivariable logistic regression models for 2PN fertilization are presented in Table 3. In Model 1, which included medium combination, patient characteristics, and semen parameters, both Group B and Group C were associated with significantly higher odds of 2PN fertilization than Group A.

**Table 3:** Multivariable logistic regression analysis for two-pronuclear (2PN) fertilization using cycle-clustered robust standard errors Values are presented as adjusted odds ratios (aORs) with 95% confidence intervals (CIs). Logistic regression models were fitted at the oocyte level, and standard errors were clustered at the oocyte retrieval cycle level to account for within-cycle correlation. Group A was used as the reference category for medium combination. Model 1 was adjusted for female age at oocyte retrieval, BMI, number of viable oocytes at retrieval, male age at oocyte retrieval, total motile sperm count per ejaculate, and medium combination. Model 2 was additionally adjusted for time from semen collection to preparation, oocyte pre-incubation time, and gamete co-incubation time. Time-related variables were analyzed per 10-minute increase. Total motile sperm count was analyzed per 10 × 10^6^ spermatozoa. The dash indicates variables not included in the model. Group A: Iso × Gx-IVF; Group B: Gra × Gx-IVF; Group C: Gra × SF; BMI: body mass index; IVF: in vitro fertilization; Iso: ISolate Stock Solution; Gra: ORIGIO Gradient 90; Gx-IVF: Gx-IVF fertilization medium; SF: Sequential Fert In group labels, the symbol × denotes the combination of the sperm preparation medium and fertilization medium.

Variable	Model 1 aOR (95% CI)	p-value	Model 2 aOR (95% CI)	p-value
Medium combination				
Group A	Reference	-	Reference	-
Group B	2.016 (1.091-3.726)	0.025	2.307 (1.165-4.572)	0.017
Group C	2.872 (1.253-6.586)	0.013	2.738 (1.001-7.484)	0.050
Female age at oocyte retrieval, per year	0.943 (0.866-1.028)	0.181	0.953 (0.871-1.043)	0.299
BMI, per 1 kg/m^2^ increase	0.974 (0.889-1.066)	0.563	0.973 (0.892-1.061)	0.536
Number of viable oocytes at retrieval, per oocyte	0.989 (0.960-1.018)	0.445	0.994 (0.967-1.023)	0.689
Male age at oocyte retrieval, per year	1.045 (0.979-1.114)	0.187	1.052 (0.990-1.117)	0.104
Total motile sperm count per ejaculate, per 10 × 10^6^ spermatozoa	1.027 (0.997-1.059)	0.076	1.032 (0.996-1.068)	0.082
Time from semen collection to preparation, per 10 min	-	-	1.033 (0.954-1.120)	0.424
Oocyte pre-incubation time, per 10 min	-	-	0.971 (0.910-1.035)	0.365
Gamete co-incubation time, per 10 min	-	-	0.966 (0.783-1.192)	0.748

In Model 2, after additional adjustment for time-related laboratory factors, Group B remained significantly associated with higher odds of 2PN fertilization than Group A (adjusted odds ratio (aOR), 2.307; 95% confidence interval (CI), 1.165-4.572; p = 0.017). Group C showed a similar trend toward higher odds of 2PN fertilization after full adjustment, although this association was borderline (aOR, 2.738; 95% CI, 1.001-7.484; p = 0.050).

Secondary analysis for 0PN

As a secondary analysis, the same multivariable modeling strategy was applied using 0PN fertilization as the dependent variable. In Model 1, both Group B and Group C were associated with significantly lower odds of 0PN fertilization than Group A.

In Model 2, after additional adjustment for time-related laboratory factors, Group B remained significantly associated with lower odds of 0PN fertilization than Group A (aOR, 0.357; 95% CI, 0.148-0.861; p = 0.022), whereas Group C showed a similar association that did not reach statistical significance (aOR, 0.243; 95% CI, 0.058-1.022; p = 0.053) (Table 4).

**Table 4:** Multivariable logistic regression analysis for 0PN fertilization using cycle-clustered robust standard errors Values are presented as adjusted odds ratios (aORs) with 95% confidence intervals (CIs). Logistic regression models were fitted at the oocyte level, and standard errors were clustered at the oocyte retrieval cycle level to account for within-cycle correlation. Group A was used as the reference category for medium combination. Model 1 was adjusted for female age at oocyte retrieval, BMI, number of viable oocytes at retrieval, male age at oocyte retrieval, total motile sperm count per ejaculate, and medium combination. Model 2 was additionally adjusted for time from semen collection to preparation, oocyte pre-incubation time, and gamete co-incubation time. Time-related variables were analyzed per 10-minute increase. Total motile sperm count was analyzed per 10 × 10^6^ spermatozoa. The dash indicates variables not included in the model. Group A: Iso × Gx-IVF; Group B: Gra × Gx-IVF; Group C: Gra × SF; BMI: body mass index; 0PN: absence of pronuclei; IVF: in vitro fertilization; Iso: ISolate Stock Solution; Gra: ORIGIO Gradient 90; Gx-IVF: Gx-IVF fertilization medium; SF: Sequential Fert In group labels, the symbol × denotes the combination of the sperm preparation medium and fertilization medium.

Variable	Model 1 aOR (95% CI)	p-value	Model 2 aOR (95% CI)	p-value
Medium combination				
Group A	Reference	-	Reference	-
Group B	0.413 (0.182-0.935)	0.034	0.357 (0.148-0.861)	0.022
Group C	0.223 (0.062-0.802)	0.022	0.243 (0.058-1.022)	0.053
Female age at oocyte retrieval, per year	1.097 (0.988-1.218)	0.083	1.087 (0.976-1.211)	0.129
BMI, per 1 kg/m^2^ increase	1.037 (0.943-1.141)	0.449	1.036 (0.946-1.134)	0.449
Number of viable oocytes at retrieval, per oocyte	1.016 (0.982-1.052)	0.365	1.012 (0.981-1.045)	0.447
Male age at oocyte retrieval, per year	0.945 (0.878-1.016)	0.127	0.939 (0.873-1.010)	0.092
Total motile sperm count per ejaculate, per 10 × 10^6^ spermatozoa	0.941 (0.891-0.993)	0.028	0.937 (0.884-0.993)	0.027
Time from semen collection to preparation, per 10 min	-	-	0.978 (0.898-1.065)	0.608
Oocyte pre-incubation time, per 10 min	-	-	1.032 (0.959-1.109)	0.403
Gamete co-incubation time, per 10 min	-	-	1.026 (0.781-1.349)	0.852

## Discussion

In this single-center retrospective cohort study, sperm preparation and fertilization medium-combination protocols were associated with fertilization outcomes during short gamete co-incubation in conventional IVF. In the unadjusted analysis, Groups B and C had higher 2PN fertilization rates and lower 0PN rates than Group A (Table 2). In the cycle-clustered multivariable analysis, Group B remained significantly associated with higher odds of 2PN fertilization after adjustment for patient characteristics, semen parameters, and time-related laboratory factors. Group C showed an association in the same direction after full adjustment, although this association was borderline (Table 3). In the secondary analysis for 0PN fertilization, Group B remained significantly associated with lower odds of 0PN, whereas Group C showed a similar association that did not reach statistical significance after full adjustment (Table 4). These findings suggest that medium-combination protocols may be related to fertilization outcomes; however, they should be interpreted as hypothesis-generating because the protocols were implemented during different laboratory periods rather than randomly assigned.

Several biologically plausible explanations may be considered, although they remain speculative. Groups B and C used ORIGIO Gradient 90, which had a higher measured pH than the 90% density gradient prepared from ISolate Stock Solution used in Group A. Previous studies have suggested that sperm preparation media may influence sperm motility, viability, and DNA integrity [15]. Sperm motility and capacitation are also closely linked to pH regulation, and acidic pH has been reported to impair human sperm motility and capacitation. In addition, capacitation-associated intracellular alkalinization has been reported in human spermatozoa [5,6]. The pH of the human female reproductive tract varies according to anatomical site, menstrual phase, and measurement conditions [7,8]. Therefore, differences in sperm preparation medium pH may have contributed to the observed associations. However, pH was not experimentally manipulated in the present study, and the independent effect of pH could not be isolated. Thus, the findings should be interpreted as associations with overall medium-combination protocols rather than evidence of a direct pH-mediated effect.

The fertilization medium may also have contributed to the observed differences. Group C used ORIGIO Sequential Fert, whereas Groups A and B used Gx-IVF. Zagers et al. reported compositional differences among commercially available human embryo culture media, including differences in glucose concentrations across fertilization media [11]. ORIGIO Sequential Fert was reported to contain 5.01 mM glucose. Glucose is relevant to sperm function because it has been reported to support human sperm motility and capacitation [9]. The presence of glucose in the medium has also been reported to be required for sperm penetration of the human zona pellucida in an experimental model [10]. Although Gx-IVF was not directly listed in the supplementary table reported by Zagers et al., Mizumoto et al. stated that Gx-series media and G-series media have identical compositions except for antioxidant supplementation [12]. Nevertheless, glucose concentrations were not directly measured in the present study, and the independent effect of fertilization medium composition could not be isolated from the overall medium-combination protocol. Therefore, interpretations related to glucose should be considered exploratory and hypothesis-generating.

An important finding was that the estimates for Group C became less robust after adjustment for time-related variables, particularly in the 0PN fertilization model (Tables 3, 4). This pattern suggests that laboratory timing factors, including oocyte pre-incubation time and gamete co-incubation time, may have contributed, at least in part, to the apparent group differences. This interpretation is consistent with the broader concept that IVF outcomes may be influenced by the culture system as a whole, including medium composition, pH, gas conditions, temperature, and handling procedures [16-18]. Previous studies and consensus recommendations have emphasized that IVF laboratory conditions and handling procedures can affect embryo development and clinical outcomes, supporting the need to interpret medium-related findings within the broader culture-system context [18-20]. In contrast, Group B remained significantly associated with higher odds of 2PN fertilization and lower odds of 0PN after full adjustment (Tables 3, 4). This finding may indicate that sperm preparation conditions, or other aspects of the Group B protocol, were associated with fertilization outcomes under short co-incubation conditions; however, causal inference cannot be established in this retrospective design.

Although Groups B and C had higher 2PN fertilization rates and lower 0PN rates than Group A, the Day 2 good-quality embryo rate did not differ significantly among the groups (Table 2). This pattern suggests that the medium-combination protocol may have been more strongly associated with fertilization than with subsequent cleavage-stage embryo morphology. Alternatively, the study may have been underpowered to detect differences in Day 2 embryo quality. Therefore, the present findings should be interpreted primarily as data on fertilization outcomes rather than as evidence of improved embryo developmental competence.

This study has several strengths. First, outcomes were evaluated per mature oocyte, and mature oocytes were explicitly defined as those with at least one polar body observed at denudation after IVF. Second, the analysis accounted for the non-independence of oocytes derived from the same treatment cycle by applying cycle-clustered robust standard errors. Third, actual laboratory timing variables, including time from semen collection to preparation, oocyte pre-incubation time, and gamete co-incubation time, were incorporated into the adjusted models.

This study also has several limitations. First, the retrospective, single-center design limits causal inference and generalizability. The medium-combination protocols were implemented during different laboratory protocol periods rather than randomly assigned. The analyzable implementation sequence was Group B, followed by Group A and then Group C, with December 2023 treated as an excluded mixed-medium transition period, although Group A was used as the analytical reference category. Therefore, calendar-time effects, laboratory workflow optimization, embryologist experience, staffing changes, equipment maintenance, quality-control variation, or other unmeasured temporal factors may have contributed to the observed associations. Because medium combination and calendar period were closely linked, residual confounding by time cannot be excluded. The retrieval year was not included in the final multivariable models because it was highly collinear with medium combination, which would have resulted in unstable or non-estimable coefficients.

Second, this study evaluated medium-combination protocols and could not isolate the independent effects of sperm preparation medium, fertilization medium, pH, glucose concentration, or their interaction. Glucose concentrations were not directly measured in the present study, and pH was measured under specific laboratory conditions rather than manipulated experimentally. Third, several potentially relevant variables, including exact abstinence duration, sperm morphology, sperm DNA fragmentation, osmolality, lot-to-lot variation, embryologist-specific effects, and detailed laboratory quality-control indicators, were unavailable. Finally, the present analysis was restricted primarily to fertilization and Day 2 embryo morphology and did not evaluate blastocyst formation, implantation, clinical pregnancy, miscarriage, or live birth. Thus, the clinical applicability of the findings remains limited.

## Conclusions

Medium-combination protocols were associated with fertilization outcomes during short gamete co-incubation in conventional IVF. In cycle-clustered multivariable analyses, Group B was consistently associated with higher odds of 2PN fertilization and lower odds of 0PN fertilization than Group A, whereas Group C showed an association in the same direction with less robust statistical evidence after full adjustment. These findings suggest that sperm preparation and fertilization medium-combination protocols may be related to fertilization outcomes in short co-incubation IVF protocols. However, the results should be interpreted cautiously because the medium combinations were implemented during different laboratory protocol periods as part of routine practice rather than assigned randomly, and downstream reproductive outcomes were not evaluated. Further prospective studies are warranted to validate these findings and to clarify which specific medium characteristics may be clinically relevant.
